# Hypoxia Changes the Expression of the Epidermal Growth Factor (EGF) System in Human Hearts and Cultured Cardiomyocytes

**DOI:** 10.1371/journal.pone.0040243

**Published:** 2012-07-05

**Authors:** Mathias Munk, Ashfaque Ahmed Memon, Jens Peter Goetze, Lars Bo Nielsen, Ebba Nexo, Boe Sandahl Sorensen

**Affiliations:** 1 Department of Clinical Biochemistry, Aarhus University Hospital, Aarhus, Denmark; 2 Department of Clinical Biochemistry, Rigshospitalet, Copenhagen, Denmark; 3 Department of Biomedical Sciences, University of Copenhagen, Copenhagen, Denmark; UAE University, Faculty of Medicine & Health Sciences, United Arab Emirates

## Abstract

**Background:**

The epidermal growth factor (EGF) receptors HER2 and HER4 and the ligands HB-EGF and NRG1 are crucial for heart development. The purpose of our study was to investigate the role of the complete EGF system in relation to hypoxia of the heart.

**Methodology/Principal Findings:**

We examined the mRNA expression by real time PCR of the 4 receptors and 12 ligands from the EGF-system in paired normoxic and hypoxic biopsies isolated from human hearts during coronary artery bypass operation. Compared to normoxic biopsies, hypoxic samples showed down-regulation of HER2 (*P = 0.0005*) and NRG1 (both α (*P = 0.02*) and β (*P = 0.03*) isoforms). In contrast, HB-EGF (*P = 0.0008*), NRG2β (*P = 0.01*) and EGFR (*P = 0.02*) were up-regulated. As HER2 is essential for heart development and we find its expression reduced under hypoxia we investigated the effect of HER2 inhibition in hypoxic HL-1 cardiomyocytes by treatment with trastuzumab (20 nM). This resulted in inhibition of cardiomyocyte proliferation, but interestingly only in hypoxic cells. Co-treatment of HL-1 cells with HB-EGF (10 nM) but not with NRG-1 (5 ng/ml) rescued the cardiomyocytes from HER2 inhibition. HL-1 cardiomyocytes exposed to hypoxia revealed nuclear translocation of activated MAPK and the activity of this downstream signaling molecule was decreased by HER2 inhibition (20 nM trastuzumab), and re-established by HB-EGF (10 nM).

**Conclusions/Significance:**

Hypoxia in the human heart alters the expression of the EGF system. Mimicking the HER2 down-regulation seen in the human heart in cultured cardiomyocytes inhibited their proliferation under hypoxic conditions. Interestingly, HB-EGF is induced in the hypoxic human hearts, and rescues hypoxic cardiomyocytes from the effect of HER2 inhibition in the *in vitro* model. The results have implications for future treatment strategies of patients with ischemic heart disease.

## Introduction

The epidermal growth factor (EGF) system plays an important role in regulating normal heart physiology during development as well as in the postnatal heart. In mice, genetic knockout of the epidermal growth factor receptors HER2 (human EGF receptor 2) and HER4 (human EGF receptor 4) or the activating ligands heparin binding-EGF like growth factor (HB-EGF) and neuregulin 1 (NRG1) lead to death *in utero* due to massive defects of the cardiac valves and myocardium [1]–[4]. Another clue to the importance of HER2 in the heart comes from observations in breast cancer patients treated with the HER2 inhibitory antibody trastuzumab. These women have an increased risk of developing cardiomyopathy especially when trastuzumab is combined with chemotherapy [5]–[8].

The EGF receptor family belongs to the receptor tyrosine kinases and consists of four receptors; EGFR (also known as ErbB1 or HER1), HER2 (Neu or ErbB2), HER3 (ErbB3), and HER4 (ErbB4) [9]. The receptors form an integrated network with at least 10 known ligands; epidermal growth factor (EGF), heparin binding-EGF like growth factor (HB-EGF), epiregulin (Epi), betacellulin (BCL), amphiregulin (AR), transforming growth factor α (TGF-α), and the neuregulins (NRG) encoded for by four genes and containing numerous splice-variants. The receptors are susceptible to ligand activation and hetero- or homo-dimerize [10]. Specific ligands activate only a subset of receptors and this forms a complex network with varied downstream signaling [9]. Ligand binding and dimerization of EGF-receptor members lead to auto-phoshorylation of the tyrosine kinase domain which in turn leads to diverse downstream signaling events including activation of pathways such as Ras/Raf/MAP kinase and phophatidylinositol-3 kinase/Akt (PI3-K/Akt).

After myocardial infarction due to plaque rupture or damage from chronic hypoxia, the heart is unable to fully reconstitute because the majority of the cardiomyocytes are terminally differentiated. Only mono-nucleated cardiomyocytes, which constitute a small fraction of all cardiomyocytes can divide [11]. The myocardium is, therefore, highly dependent on cell survival mechanisms to tolerate acute or chronic hypoxia. The EGF-system plays an important role in survival mechanisms [12]. Especially EGFR and HER2 are known for their capabilities to phosphorylate the PI3-K/Akt and Ras/Raf/MAPK pathways resulting in cell survival. MAPK has been implicated in cell-survival through activation of the 90-kDa ribosomal S6 kinases (RSK1–4) which inactivates the pro-apoptotic factor BAD and activate the survival factor nuclear factor-κB, thus promoting cell-survival [13]. MAPK also activates the nuclear protein hypoxia inducible factor 1 (HIF-1) which is involved in essential processes related to adaption to ischemia [14], [15]. Akt can, when phosphorylated under normoxic conditions, down-regulate the pro-apoptotic factors caspase-9 and BAD, via BCL2 family members, and up-regulate the survival factors nitric oxide and nuclear factor-κB, thereby promoting cell survival [16], [17]. Under hypoxic conditions however, current data suggests that Akt functions oppositely by causing necrosis due to PI3-K mediated changes in glucose metabolism [18], [19].

How cardiomyocytes utilize the EGF-system during hypoxia is not determined. The pre-form of HB-EGF, proHB-EGF is highly expressed in the heart and also functions as a diphtheria toxin receptor explaining why diphtheria toxins can induce myocarditis [20]. In animal models, HB-EGF is up-regulated after myocardial infarction and involved in cardiac remodeling by activating non-cardiomyocytes [21]–[23]. Recombinant NRG-1 improves cardiac functions and survival in various experimental models of cardiomyopathy, including cardiomyopathy due to ischemia [24].

In the present study, we explore the regulation of the complete EGF-system (all four receptors and their activating ligands) following myocardial hypoxia in the human heart. We show that hypoxia *in vivo* down-regulates the mRNA expression of HER2 and both the α and β isoforms of NRG1, while EGFR and its activating ligand HB-EGF is up regulated, as is NRG2β. Employing a cardiomyocyte *in vitro* model we demonstrate that HER2 inhibition is particularly inhibitory for cardiomyocyte proliferation under hypoxic conditions and that this effect can be diminished by treatment with HB-EGF.

## Materials and Methods

### Ethics Statement

All patients gave informed written consent and the protocol with the file number KF 01-101/99 was approved by the local ethics committee (the ethics committee of Copenhagen and Frederiksberg). The pig samples came from the Steff-Houlberg Slaughterhouse located in Ringsted, Denmark.

### Human Biopsies from Patients Undergoing Coronary Artery Bypass Graft Operation

Ten patients admitted for coronary artery bypass graft (CABG) with diagnosed three-vessel disease were included as described [25]. Pre-operatively, all developed pectoral angina during exercise stress tests. A normoxic biopsy from the left atrium (control) and a hypoxic biopsy from the left ventricle were obtained. Oxygen tensions were measured using a REVOXODE pO_2_ microprobe electrode (Harvard Apparatus, Holliston, MA) connected to a LiCOX CMP instrument (Plainsboro, NJ). The microprobe was sutured to the atrial and ventricular wall to verify normoxia and hypoxia. Specimens (10–30 mg) were sampled from an area immediately adjacent to the probe using either a scalpel or a 5 mm muscle biopsy cannula (Still, Stockholm, Sweden). The biopsies were directly frozen in liquid nitrogen and stored at -141°C.

### Pig Biopsies for Examination of Regional Variability in mRNA Expression in the Heart

Twelve Danish-bred pigs were used and biopsies from the heart used to examine for any regional variability in mRNA expression. The pigs were killed and transmural biopsies (0.5–1.0 g) were obtained from the left atrium, posterior wall of the left ventricle, and the anterior wall of the left ventricle. Biopsies were immediately frozen in liquid nitrogen or placed on dry ice. One pig was excluded due to inadequate mRNA quality for analysis.

### RNA Analysis of HL-1 Mouse Cardiomyocytes Exposed to Hypoxia

HL-1 cells were a kind gift from Dr. William Claycomb (Department of Biochemistry & Molecular Biology, School of Medicine, New Orleans) [26]. HL-1 cells were grown in Claycomb medium containing 10% FBS, Penicillin/Streptomycin 100 U/ml and 100 µg/ml respectively, 0.1 mM Norepinephrine, and 2 mM L-Glutamine. Cell culture flasks were pre-coated with a solution of 0.5% fibronectin and 0.2% gelatin overnight at 37°C. Cells were routinely checked for mycoplasma infections by a DNA staining method employing a DNA-binding fluorochrome. Conditioned medium from the HL-1 cells was added to cultured Vero cells. Samples were incubated, stained, and the presence of mycoplasma infection was examined using fluorescent microscopy. The HL-1 cells were found not to be mycoplasma infected.

For 4, 24 and 72 hour hypoxia experiments, 400.000, 300.000, and 150.000 cells respectively were seeded in 28-cm^2^ glass petri dishes in 3 ml medium.

### mRNA Purification and cDNA Amplification

Total RNA was isolated using TRIzol (Invitrogen) or the RNeasy mini kit for fibrous tissue (Quiagen, Chatsworth, CA). Both TRIzol and RNeasy kits were used following the manufacturer’s instructions. Total RNA was DN’ase treated twice with 10 U RNAase-free DN’ase I (Promega, Madison, USA) for 15 minutes at room temperature to ensure no DNA contamination. First-strand cDNA was synthesized at 37°C from 0.1 µg RNA with M-MULV reverse transcriptase (40 U, Roche A/S, Copenhagen, Denmark) and OligoT primers.

The best-suited reference gene was determined using Normfinder [27]. For normalization of human samples, Beta-2-microglobulin, Beta-actin, and Glyceraldehyde 3-phosphate dehydrogenase (GAPDH) were examined and Beta-actin was found to be the most stable reference gene. For normalization of pig samples, Beta-actin, GAPDH, ribosomal protein L4 (RPL-4), and hypoxanthine phosphoribosyltransferase 1 (HPRT-1) were examined and GAPDH was found to be the most stable reference gene. For normalization of HL-1 mouse cardiomyocytes Beta-2-microglobulin, hydroxymethylbilane synthase (HMBS), and HPRT-1 were examined and Beta-2-microglobulin was chosen as the most stable reference gene.

### Real-time RT-PCR mRNA Analysis of the EGF-system

All primers, calibrators, and conditions are shown in table 1. Quantitative real-time PCR analyses were performed using a Lightcycler 480 with Faststart SYBR Green mix and 96 well plates (all from Roche). Total RNA either from different cell lines or from a pool of total RNA from pig hearts was used as calibrators (see table 1).

**Table 1:** Complete list of the RT-PCR primers, concentrations, annealing temperatures, amplicon sizes, and cell lines or RNA used as calibrators in this study*.

Assay	Forward	Reverse	Primer conc.Annealing temp	Amp. size	Calibrator
**Human Assays**					
EGFR	5′-GAG AAC GCC TCC CTC A-3′	5′-GGT ACT CGT CGG CAT C-3′	5pmol 54°C	261bp	HCV
HER2	5′-AGA TGT TCG GCC CCA GCC CCC TT-3′	5′-GTG GAG CCC CCC GCT CTG GTG-3′	10pmol 68°C	272bp	HCV
HER3	5′-GGT GCT GGG CTT GCT TTT-3′	5′-CGT GGC TGG AGT TGG TGT TA-3′	5pmol 65°C	365bp	HEC
HER4 CYT1	5′-GAT GAT CGT ATG AAG CTT CCC A-3′	5′-AGG AGG AGG GCT GTG TC-3′	2.5pmol 60°C	221bp	CYT1 RNA
HER4 CYT2	5′-GAT GAT CGT ATG AAG CTT CCC A-3′	5′-CGG TAT ACA AAC TGG TTC CTA TTC-3′	5pmol 60°C	194bp	CYT2 RNA
HER4 JM	5′-CAG TGT GAG AAG ATG GAA GAT G-3′	5′-CCT TTT GAT GAT CTT CCT TCT AAC-3′	5pmol 58°C	375/346bp	RT4
EGF	5′-GAC TTG GGA GCC TGA GCA GAA-3′	5′-CAT GCA CAA GTG TGA CTG GAG GT-3′	5pmol 66°C	90bp	KLE
HB-EGF	5′-GGT GGT GCT GAA GCT CTT TC-3′	5′-CCC CTT GCC TTT CTT CTT TC-3′	5pmol 61°C	282bp	HCV
TGF-α	5′-GCC CGC CCG TAA AAT GGT CCC CTC-3′	5′-GTC CAC CTG GCC AAA CTC CTC CTCTGG G-3′	5pmol 70°C	528bp	HCV
Epiregulin	5′- AAA GTG TAG CTC TGA CAT G-3′	5′-CTG TAC CAT CTG CAG AAA TA-3′	10pmol 60°C	238bp	HCV
Betacellulin	5′-TCT AGG TGC CCC AAG C-3′	5′-GTG CAG ACA CCG ATG A-3′	5pmol 66°C	221bp	KLE
Amphiregulin	5′-GGC TCA GGC CAT TAT GC-3′	5′- ACC TGT TCA ACT CTG ACT GA-3′	10pmol 58°C	266bp	HCV
NRG1-α	5′-ATC CAC CAC TGG GAC A-3′	5′-TTT GGA TCA TGG GCA-3′	5pmol 60°C	179bp	KLE
NRG1-β	5′-TAG GAA ATG ACA GTG CCT C-3′	5′-CGT AGT TTT GGC AGC GA-3′	5pmol 65°C	321bp	KLE
NRG2-α	5′-AAA TAT GGC AAC GGC AG-3′	5′-CGC AAA GGC AGT TTC T-3′	5pmol 60°C	308bp	KLE
NRG2-β	5′-GCT TTA CGT CAA CAG CG-3′	5′-CCG GTG TAT CCC ACA G-3′	5pmol 63°C	236bp	HCV
NRG3	5′-ACA GTG CAA GCG AAA AC-3′	5′-CAC TAT GAT ATG AGG GCG-3′	5pmol 61°C	256bp	KLE
NRG4	5′-CTG TTG TCT GCG GTA TTC-3′	5′-TCA TTC TTG GTC AAG AGA GT-3′	5pmol 61°C	107bp	RT4
beta-actin	5′-GGC GGC ACC ACC ATG TAC CCT-3′	5′-AGG GGC CGG ACT CGT CAT ACT-3′	10pmol 68°C	202bp	HCV
**Pig Assays**					
EGFR	5′-CCT TGG GAA CTT GGA GAT CA-3′	5′-GGT TTT ATT GGC CCC GTA GT-3′	5pmol 60°C	208bp	Total pig RNA
HER2	5′-GGT GTA GGC TCC CCG TAT GT-3′	5′-GCA ATC TGC ACA CAC CAG TT-3′	5pmol 60°C	164bp	Total pig RNA
HB-EGF	5′-GGT GGT GCT GAA GCT CTT TC-3′	5′-CTC AAA AGG TCC AGG TCT GC-3′	5pmol 60°C	201bp	Total pig RNA
Beta-actin	5′-CAC GCC ATC CTG CGT CTG GA-3′	5′-AGC ACC GTG TTG GCG TAG AG-3′	5pmol 60°C	100bp	Total pig RNA
GAPDH	5′ACA CTC ACT CTT CTA CCT TTG-3′	5′-CAA ATT CAT TGT CGT ACC AG-3′	5pmol 57°C	90bp	Total pig RNA
**Mouse Assays**					
EGFR	5′-GAG AGC GCC TTC CAC-3′	5′-GAT ACT CAT CAG CAT-3′	5pmol 54°C	261bp	HL-1
HER2	5′-AGA GGT TCG GCC TCA-3′	5′-GTG GAG GAC CCT GCT-3′	5pmol 60°C	272bp	HL-1
HER3	5′-GGT GCT GGG TTT CCT TCT-3′	5′-CAT GGC TGG AGT TGG TAT TG-3′	5pmol 60°C	365bp	HL-1
HER4	5′-TGA ACA ATG TGA TGG CAG GT-3′	5′-TGA AGT TCA TGC AGG CAA AG-3′	5pmol 60°C	116bp	HL-1
HB-EGF	5′-GGT GAT GCT GAA GCT CTT TC-3′	5′-CCC CTT TCC TTT CTT CTT CT-3′	5pmol 60°C	283bp	HL-1
NRG1-α	5′-ATC CAC GAC TGG GAC C-3′	5′-TGT AGA AGC TGG CCA TTA-3′	10pmol 54°C	179bp	HL-1
NRG2-β	5′-ACT CCA TGT CAA CAG CG-3′	5′-CCG GTG TAT CCC ACA G-3′	5pmol 60°C	236bp	HL-1
BNP	5′-CTG AAG GTG CTG TCC CAG AT-3′	5′-GTT CTT TTG TGA GGC CTT GG-3′	5pmol 60°C	199bp	HL-1
β-2-microglobulin	5′-ATT CAC CCC CAC TGA GAC TG-3′	5′-TGC TAT TTC TTT CTG CGT GC-3′	5pmol 60°C	193bp	HL-1

* Human HER2 assay uses a taqman® probe: Fam 5′ - CAG ATT GCC AAG GGG ATG AGC TAC CTG –3′ Tamra (10 pmol).

### Fluorescent Microscopy

HL-1 cells (n = 5000) were seeded in fibronectin/gelatin coated chamberslides and grown in 21% and 1% oxygen respectively for 72 hours. Cells were immediately fixed in Lillys Fluid (VWR, Bie & Berntsen, Denmark) for 20 minutes. The cells were washed in PBS containing 0.1% Triton X-100 and 5% BSA. Slides were blocked for 1 hour with 0.03% Triton and 0.5% BSA in Dulbecco PBS and treated over night at 4°C with anti-p-MAPK (Cell Signaling 9101 1:1000). Alexa 594 conjugated secondary donkey anti-rabbit antibody was then treated for 40 minutes at 1∶4000. After washing the slides were mounted with Prolong® Gold anti-fade with DAPI (Invitrogen). Cells were visualized using an inverted Zeiss Axiovert 200 fluorescent microscope at 100X magnification with oil. Images were obtained using Zeiss MR3 monochrome camera.

**Figure 1:**
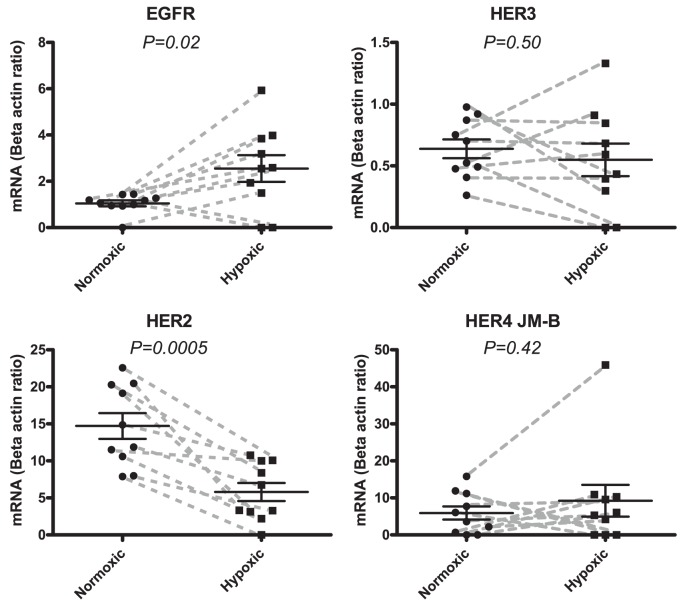
mRNA expressions of the EGF receptors EGFR, HER2, HER3, and the JM-b isoform of HER4 in human biopsies. Samples were obtained from a normoxic part of the heart and compared to another sample from the hypoxic part of the same heart. Expressions of mRNA are adjusted by division with the expression of beta-actin. Normoxic tissue expressions are depicted with circular points and hypoxic expression with squares. Each paired sample is connected with a grey dashed line. Data is represented with means and SEM. EGFR shows significant up-regulation from a mean value of 1.0 (normoxic) to a mean value of 2.6 (mean of hypoxic) (*P = 0.03*). HER2 shows significant down-regulation from 15.0 ((mean of normoxic) to 5.8 ((mean of hypoxic) (*P = 0.0005*). The differences in HER3 and HER4/JM-b are non-significant (*P = 0.5* and *P = 0.4,* respectively).

**Figure 2:**
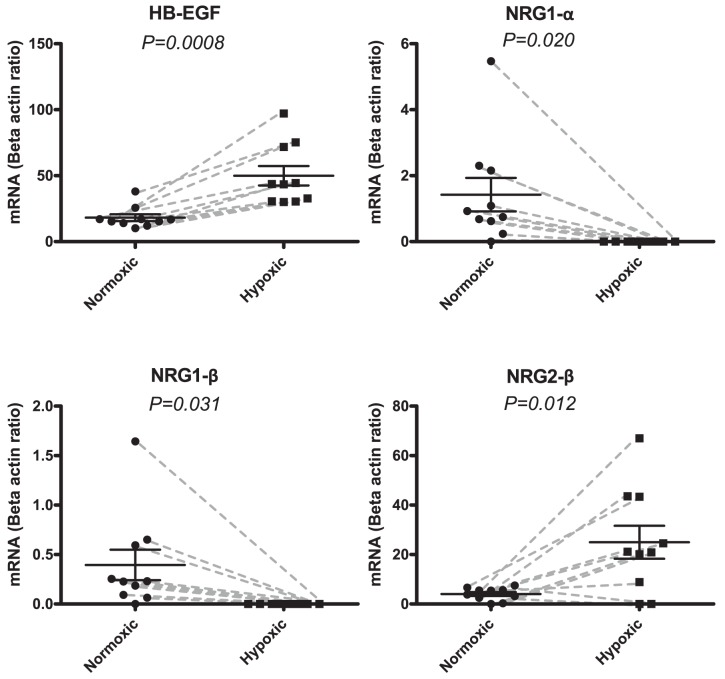
mRNA expressions of the ligands HB-EGF, NRG1-α, NRG1-β, and NRG2-β in the human biopsies. Samples were obtained from a normoxic part of the heart and compared to a hypoxic part. Normoxic tissue expressions are depicted with circular points and hypoxic expression with squares. Each paired sample is connected with a grey dashed line. Data is represented with means and SEM as the ratio between the reference gene beta actin. HB-EGF shows significant up-regulation from a mean value of 18.1 (Normoxic) to 50.0 (Hypoxic) (*P = 0.0008*). NRG1-α shows significant down-regulation from a mean value of 1.4 (Normoxic) to 0.0 (Hypoxic) (*P = 0.0204*). NRG1-β shows significant down-regulation from means 0.4 (Normoxic) to 0.0 (Hypoxic) (*P = 0.0308*). NRG2-β shows significant up-regulation from means 4.1 (Normoxic) to 25.0 (Hypoxic) (P = 0.0120).

### Western Blot Analysis of HL-1 Cardiomyocytes Exposed to Hypoxia

HL-1 mouse cardiomyocytes were grown on fibronectin/gelatin coated glass petri dishes and placed in a sealed growth chamber and perfused with a water saturated air mixture containing 1% oxygen, 5% CO_2_, and 94% nitrogen for 1 or 2 hours at 37°C. Samples were treated with either 20 nM trastuzumab (Roche, Denmark) all through the experiment and/or 10 nM HB-EGF (R&D systems, England). HL-1 cells grown at 21% oxygen, 5% CO_2_, and 74% nitrogen were used as control. Immediately after termination of the experiment the cells were washed in ice-cold PBS and scraped on ice in a scraping buffer containing 4 mM iodoacetate, 1 mM sodium-orthovanadate, and 1 µg/ml of the following protease inhibitors; pepstatin, chymostatin, leupeptin, and aprotinin. After scraping, the cells were centrifuged at 2000 g for 3 minutes and the scraping buffer was removed. Subsequently, the cell pellet was lysed with RIPA buffer [28]. Briefly, the cells were incubated for 15 minutes on ice and centrifuged at 20.000 g for 10 minutes. The supernatant was frozen at −80°C until further analysis. Protein concentration was determined using the BCA kit (Pierce). Samples were loaded on precast 12% polyacrylamide gels (Biorad) with 50 µg protein in each lane. Proteins in the gels were transferred to a PVDF membrane and blocked for 1 hour in 5% non-fat dry milk in TBS-T (25 mM Tris, pH 7.5, 150 mM NaCL, 0.05% Tween-20). The following antibodies and concentrations were used over night at 4°C; EGFR (Cell Signaling 2232 1:1000), p-EGFR (Santa Cruz sc-12351 1:200), HER2 (Santa Cruz sc-284 1:200), p-HER2 (Millipore 06-229 1:1000), HER3 (Epitomics 1186-1 1:500), p-HER3 (Epitomics 2526-1 1:1000), HER4 (Epitomics 2218-1 1:1000), p-HER4 (Epitomics 2346-1 1:1000), Akt (Cell Signaling 9272 1:500), p-Akt (Cell Signaling 9271L 1∶500), MAPK (Cell Signaling 9102 1:1000), p-MAPK (Cell Signaling, 9101S 1∶1000), and Actin (Sigma A-5316 1:8000). Secondary HRP-cunjugated anti-mouse and anti-rabbit antibodies (Dako, Denmark) were subsequently added for 40 minutes. After washing, membranes were dipped in ECL (Amersham Biosciences) for 2 minutes and immunoreactive bands were detected by a Biospectrum(R) AC Imaging System (UVP) or by X-ray, and the individual bands were quantified with ImageJ software.

### CellTiter 96® Proliferation Assay of HL-1 Cardiomyocytes Exposed to Hypoxia and Treated with Trastuzumab, HB-EGF, and NRG-1β

5000 HL-1 cells were seeded in gelatin/fibronectin coated flat-bottomed 96 well plates overnight (Nunc, Denmark). Cells were treated with either 10 nM HB-EGF, 5 ng/ml NRG1β (R&D systems, England), 20 nM trastuzumab or a combination of 20 nM trastuzumab and 10 nM HB-EGF or 5 ng/ml NRG1β. The cells were then exposed to water saturated 1% oxygen and 21% oxygen respectively in a sealed chamber for 48 hours. Cell proliferation was then examined using CellTiter 96® AQeous Solution Cell Proliferation Assay (Promega, Wisconsin, USA) according to the manufacturer’s instructions. After incubation the plates were read on a Multiskan Ascent ELISA reader (Thermo-scientific, Waltham, USA) at 492 nM.

### Statistical Analysis

Data is represented as mean ± SE. To compare paired samples, paired t-test was used. In all other statistical analyses the non-parametric Mann-Whitney t-test was used. P-values <0.05 were considered statistically significant.

## Results

### Hypoxia Changes Ligand and Receptor Expression of the EGF-system in the Human Heart

Biopsies from 10 patients undergoing coronary bypass operation were investigated. All patients had 3-vessel disease and we compared the mRNA expression of the hypoxic part of the left ventricle to a normoxic part of the heart (left atrium) in each patient. HER2 was down regulated in the hypoxic part of the heart ((Figure 1) *P = 0.0005*). In contrast, EGFR as well as its activating ligand HB-EGF were significantly up regulated (EGFR: *P = 0.02* (Figure 1), *HB-EGF: P = 0.0008* (Figure 2)). The HER3 and the HER4 receptors were unaffected by hypoxia (Figure 1). The HER4 JM-b isoform, which is the predominant isoform in the heart, was measured [29]. Analyzing each of the two HER4 JM-b isoforms (JM-b/CYT1 and JM-b/CYT2) separately showed that they were unaffected (data not shown). Among the neuregulins (NRG1–4) the two isoforms of NRG-1 (α and β) were expressed in all but 1 patient in the normoxic part of the heart but were undetectable in all samples of the hypoxic part of the heart ((figure 2) *P = 0.02* (NRG-1α) and *P = 0.03* (NRG-1β)). In contrast, NRG2-β was up regulated in the hypoxic part of the heart (*P = 0.01*). The remaining ligands Epi, EGF, BCL, TGF-α, AR, NRG2α, NRG3 and NRG4 showed no significant changes (data not shown).

**Figure 3:**
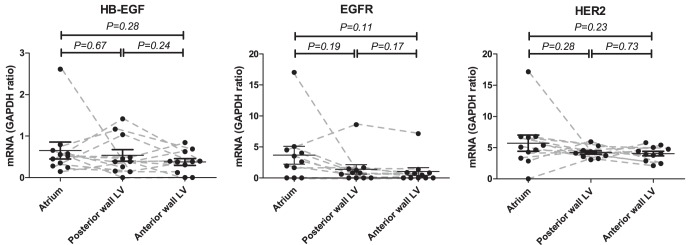
Regional expression of HB-EGF, EGFR, and HER2 in normal pig hearts. Tissue mRNA expressions are all depicted with circular points. Each paired sample is connected with a grey dashed line. Samples were obtained from the left atrium, the posterior wall of the left ventricle, or the anterior wall of the left ventricle. Data is represented with means and SEM as the ratio between the reference gene GAPDH. HB-EGF, EGFR and HER2 showed no significant regional variation in mRNA expression (all *P>0.05*).

**Figure 4:**
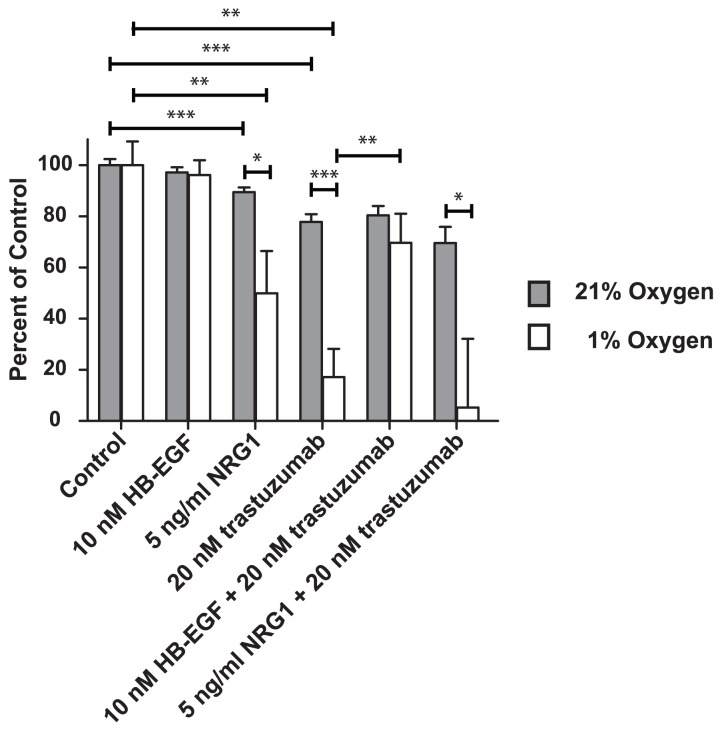
Proliferation of HL-1 cardiomyocytes upon treatment with HB-EGF or NRG1. Influence of trastuzumab inhibition. All results are shown as a percent of control with mean and SEM. Grey bars are cells grown at normoxic conditions (21% oxygen) and white bars are cells grown at hypoxic conditions (1% oxygen). There are no significant differences when treating with 10 nM HB-EGF as compared to control (*P = 0.4005* (Normoxia) and *P = 0.7282* (Hypoxia). There are significant differences when treating with NRG1β compared to control (*P = 0.0034* (Normoxia) and *P = 0.0246* (Hypoxia)) and the difference under hypoxic conditions is greater than under normoxic conditions (*P = 0.0324*). Cellular proliferation in the presence of 20 nM trastuzumab is significantly different from control (*P = 0.0002* (Normoxia) and *P = 0.0012* (Hypoxia)) but the difference is more pronounced in hypoxic cardiomyocytes compared to normoxic (*P = 0.0006*). Only under hypoxic conditions and trastuzumab treatment, can cardiomyocyte proliferation be rescued with 10 nM HB-EGF (*P = 0.5915* (Normoxia) and *P = 0.0068* (Hypoxia)), but this rescuing effect is not observed for NRG1β (*P = 0.5737* (Normoxia) and *P = 0.7130* (Hypoxia)). The inhibitory effect of combined NRG1β and trastuzumab treatment is greater in hypoxic compared to normoxic cells (*P = 0.049*). This experiment was repeated twice giving similar result.

The human samples were obtained from the left ventricle (hypoxic sample) and the left atrium (normoxic control). We investigated if the significant changes in mRNA expression observed could be due to regional differences in mRNA expression in the heart. Samples from 11 pigs were investigated. Importantly, no differences were found in expression of HB-EGF, EGFR, and HER2 between the left atrium and either the posterior or the anterior wall of the left ventricle (all *P>0.05*) (Figure 3). In the human biopsies the hypoxic sample was taken in the ventricle and the normoxic sample was taken in the atrium. Although not significant, there was a tendency towards a down-regulation of the EGFR expression in both positions of the ventricle compared to the atrium of the pig hearts. However, this further substantiates the results from the human samples where an up-regulation was found in the hypoxic ventricle as compared to the normoxic atrium, despite the tendency to a lower EGFR mRNA expression in the ventricle than in the atrium of the pig hearts. These results support that regional differences in the expression of HB-EGF, EGFR, and HER2 was not the cause of our findings in the human heart.

**Figure 5:**
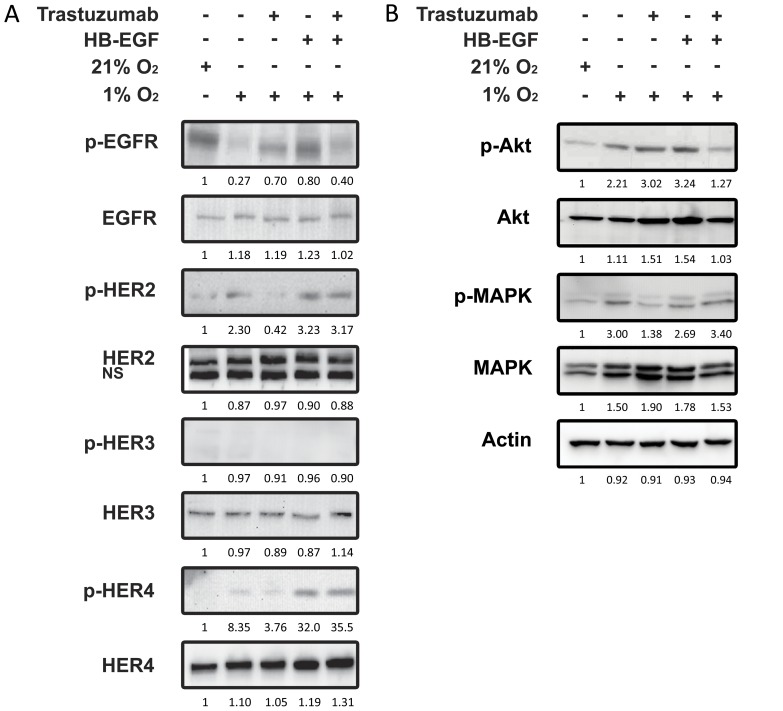
Western Blot showing HL-1 cardiomyocytes exposed to 1% oxygen with or without treatment with trastuzumab. **Influence of HB-EGF.** Mouse HL-1 cardiomyocytes kept at 1% oxygen were treated either with or without 20 nM trastuzumab for 1 hour followed by treatment with 10 nM HB-EGF for 10 minutes. Whole cell lysates were investigated for phosphorylated and total amounts of the receptors EGFR, HER2, HER3, and HER4. Also examined was the phosphorylation and total amounts of the down-stream signaling molecules MAPK and Akt. Actin was used a loading control. This experiment was repeated twice giving similar results.

### Inhibition of HER2 in HL-1 Cardiomyocytes Inhibits Proliferation which can be Counterbalanced by HB-EGF but not NRG1β

The changes in expression of the EGF system led us to investigate their role for the proliferation of hypoxic cardiomyocytes using the HL-1 cardiomyocyte cell line. To mimic the HER2 down-regulation found in the human samples we used the HER2 inhibitor trastuzumab. As shown in Figure 4, we found that treatment with 20 nM trastuzumab for 48 hours reduced the growth of the HL-1 cells under both normoxic and hypoxic conditions (*P = 0.0002* and *P = 0.001* respectively). However, when the HL-1 cells were stressed with hypoxia, the effect of HER2 inhibition was considerably higher than under normoxic conditions (*P = 0.0006*). Growth of the cells was reduced to 20% under hypoxic conditions as compared to 80% under normoxic conditions. HB-EGF was induced in the hypoxic human biopsies and most interestingly we find that 10 nM HB-EGF rescued hypoxic HL-1 cells from trastuzumab-induced growth inhibition (*P = 0.007*). This cell rescue was not found for HB-EGF in normoxic cells (*P = 0.9*). Opposite to HB-EGF NRG1 (both the α and β isoforms) was down regulated in the hypoxic human samples and NRG1β (5 ng/ml) was not capable of rescuing hypoxic HL-1 cells from trastuzumab induced inhibition of proliferation (*P = 0.7*). The inhibitory effect of combined NRG1β and trastuzumab treatment was greater for cells exposed to hypoxic as compared to normoxic conditions (*P = 0.05*). Treating cells with NRG1β alone decreased cell growth under both normoxic and hypoxic conditions (*P = 0.003* and *P = 0.02, respectively*). However, when stressed with hypoxia, NRG1β treatment increased the inhibitory effect on HL-1 cells compared to cells grown under normoxic conditions (*P = 0.03*).

**Figure 6:**
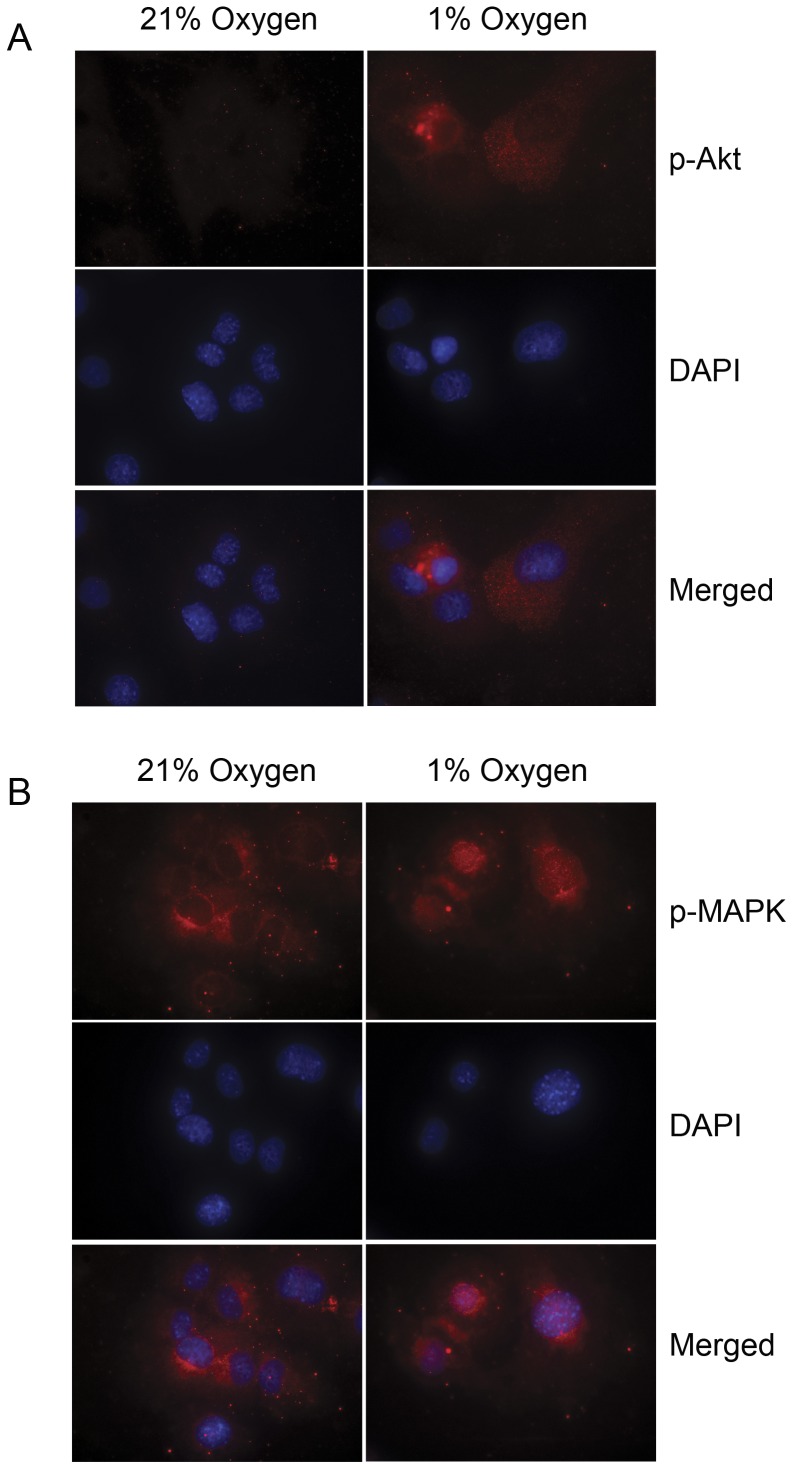
Fluorescent microscopy of phosphorylated MAPK and Akt in HL-1 cardiomyocytes exposed to 21% or 1% oxygen. A. HL-1 cardiomyocytes were exposed to 1% oxygen and compared to control cells grown in 21% oxygen for 72 hours**.** Akt reveals increased phosphorylation but no nuclear translocation of phosphorylated AKT. B. Phosphorylated MAPK reveals nuclear translocation in the hypoxic cells compared to the normoxic cells.

### HB-EGF Increases the Proliferation of HL-1 Cardiomyocytes when HER2 is Inhibited

The finding that HB-EGF rescued the hypoxic cardiomyocytes from the inhibitory effect of the HER2 inhibitor trastuzumab on proliferation led us to examine the signaling processes associated with this mechanism. HL-1 cardiomyocytes were exposed to hypoxia, treated with trastuzumab, and the effect of HB-EGF on activation of the four EGF receptors as well as the down-stream signaling molecules Akt and MAPK was analyzed. As shown in Figure 5, 1 hour of hypoxia inhibited EGFR, while HER2 and HER4 were activated (Lane 2). No activity could be detected in HER3 throughout the experiment. In hypoxic cells HER2 activation was inhibited by 20 nM trastuzumab (lane 3). HER4 and EGFR activity was unaffected by HER2 inhibition. Adding 10 nM HB-EGF to the hypoxic cells activated EGFR and HER4 as expected based on the receptor specificity of this ligand (lane 4). However, when adding HB-EGF (10 nM) together with trastuzumab (10 mM) HER2 activation was not inhibited by trastuzumab (lane 5). In contrast, the EGFR activation caused by HB-EGF was inhibited by trastuzumab. The phosphorylation of HER4 was increased significantly by HB-EGF and the activity of HER4 was unaffected by trastuzumab. This demonstrates that in hypoxic cardiomyocytes treated with the HER2 inhibitor trastuzumab HB-EGF activates HER2 and HER4, and both receptors hereby become resistant to trastuzumab inhibition. Examining the down-stream signaling, HL-1 cells exposed to hypoxia revealed increased MAPK phosphorylation (lane 2) and this activation was prevented by trastuzumab mediated HER2 inhibition (lane 3), but reactivated by HB-EGF regardless of HER2 inhibition (lane 4 and 5). Hypoxia caused an increase in Akt phosphorylation that was not affected by trastuzumab (lane 2 and 3). However, when trastuzumab was combined with HB-EGF, Akt phosphorylation was decreased (lane 4 and 5). After 2 hours of hypoxia this was even more pronounced and at this time-point HB-EGF treatment alone decreased the phosphorylation of Akt (data not shown).

### Hypoxia Induces Nuclear Translocation of Phosphorylated MAPK in HL-1 Cardiomyocytes

Under hypoxic conditions activated MAPK can translocate to the nucleus [30], and we investigated whether the activated forms of Akt and MAPK observed in the hypoxic cardiomyocytes also translocates to the nucleus. Nuclear translocation was not observed for Akt that increased its phosphorylation in HL-1 cardiomyocytes exposed to hypoxia for 72 hours but remained in the cytoplasm or membrane under both normoxic and hypoxic conditions (Figure 6A). Interestingly, exposure of HL-1 cardiomyocytes to hypoxia resulted in translocation of the phosphorylated MAPK to the nucleus (Figure 6B). In contrast, when cardiomyocytes were grown under normoxic conditions phosphorylated MAPK did not enter the nucleus.

## Discussion

We examined the changes in expression of the EGF system in the hypoxic part of the heart of patients admitted for coronary artery bypass graft. Comparing samples from the left ventricle (verified as hypoxic) with the left atrium (verified as normoxic) we observed a down-regulation of the HER2 receptor while EGFR was induced. The activating ligands HB-EGF and NRG2β were induced, while NRG1 was down-regulated. Interestingly, among the differentially expressed components from the EGF system both the HER2 receptor and the ligands HB-EGF and NRG1 are known to have an effect on the embryonic development of the heart [3], [4], [31]. We further explored the biological importance of the changes observed in the expression of HER2 in the human hypoxic cardiac tissue by using a cellular cardiomyocyte model, the mouse HL-1 cell line. This cell line is derived from cardiomyocytes and has been demonstrated to retain the beating phenotype and protein characteristics of this cell type [26]. By mimicking HER2 down-regulation with a HER2 inhibitor, we demonstrated that this inhibits cardiomyocyte proliferation. In the human hypoxic hearts, HB-EGF was induced and we found that exogenously added HB-EGF reactivated HER2 and reduced the inhibitory effect on cardiomyocyte proliferation caused by HER2 inhibition. Interestingly, this effect of HB-EGF was only seen under hypoxic conditions. This suggests a model in which the reduction in HER2 expression in the hypoxic hearts is counterbalanced by increased HB-EGF expression. Western blotting with phosphospecific antibodies shows that HB-EGF re-activates both HER2 and HER4 in the cardiomyocytes treated with trastuzumab suggesting these receptors to be involved in mediating the survival signal. The hypoxic heart biopsies showed down-regulation of HER2 and up-regulation of EGFR mRNA, whereas the HL-1 cells exposed to hypoxia decreased EGFR phosphorylation and increased HER2 phosphorylation. These apparently contradictory findings might be explained by the prolonged and more complex situation in the patients with chronic hypoxia in the heart as compared to HL-1 cells growing under well-defined conditions and exposed to hypoxia for only a short period of time. Furthermore, the contradictory results could arise as it is not possible to make a direct comparison of mRNA expression and protein phosphorylation data.

The down-stream signaling molecules MAPK and Akt are known to be influenced by EGF receptors and to be involved in cell survival mechanisms [32], [33]. We demonstrate activation of both Akt and MAPK in hypoxic cells compared to normoxic cells. Furthermore, we find nuclear translocation of activated MAPK (Figure 6), which is a mechanism that has previously been shown to be necessary for initiating cell-survival functions [30]. Nuclear MAPK is believed to be important for the cellular response to hypoxia as it is known to induce stabilization of HIF1α which is crucial for the ability of the cell to prevent hypoxia induced cell death [14], [15], [30]. Inhibition of HER2 in hypoxic cardiomyocytes resulted in a reduced activity of MAPK, but interestingly HB-EGF was also able to reactivate MAPK. In contrast, HER2 inhibition did not affect Akt activity and in contrast to its effect on MAPK HB-EGF inhibited Akt. The Akt inhibition induced by HB-EGF may be related to a special function of the PI3-K/Akt pathway during hypoxia. Aki et al found that the PI3-K/Akt pathway, under hypoxic conditions, controls the glucose-metabolism of cardiomyocytes and increased Akt signaling induce necrosis whereas decreased Akt signaling induce proliferation [18], [19]. In our study we also find HB-EGF induced MAPK stimulation even when HER2 is inhibited supporting the important functions of HB-EGF in cardiomyocytes during hypoxia.

NRG1 was reduced in the human hypoxic hearts and did not display the stimulatory effects of HB-EGF on cardiomyocyte proliferation, when HER2 was inhibited. On the contrary NRG1 reduced the proliferation of the cardiomyocytes *in vitro* under hypoxic conditions. This suggests that down-regulation of NRG1 in the human hypoxic hearts might protect cardiomyocytes during hypoxia. The roles of NRG1 in cardiac diseases are however controversial. Some clinical studies of heart failure patients, find NRG1 to be up-regulated [34]. In addition, NRG1β has been linked to disease severity in chronic heart failure where high expression of NRG1β increases the risk of receiving a heart transplant [35].

In conclusion, the results support that hypoxic heart disease affects the expression of key genes in the EGF system in cardiac myocytes. In vitro studies indicate that the changes in the EGF system influence proliferation of the cardiomyocytes. Most remarkably we show an inhibitory effect of the reduced HER2 expression, but also a beneficial effect of HB-EGF on the proliferation of hypoxic cardiomyocytes. This observation suggests the possibility that there may be a clinical benefit of treating patients with acute hypoxia with HB-EGF but further studies are needed before this can be concluded.

## References

[1] Lee KF, Simon H, Chen H, Bates B, Hung MC (1995). Requirement for neuregulin receptor erbB2 in neural and cardiac development.. Nature.

[2] Gassmann M, Casagranda F, Orioli D, Simon H, Lai C (1995). Aberrant neural and cardiac development in mice lacking the ErbB4 neuregulin receptor.. Nature.

[3] Iwamoto R, Yamazaki S, Asakura M, Takashima S, Hasuwa H (2003). Heparin-binding EGF-like growth factor and ErbB signaling is essential for heart function.. Proc Natl Acad Sci U S A.

[4] Meyer D, Birchmeier C (1995). Multiple essential functions of neuregulin in development.. Nature.

[5] Suter TM, Procter M, van Veldhuisen DJ, Muscholl M, Bergh J (2007). Trastuzumab-associated cardiac adverse effects in the herceptin adjuvant trial.. J Clin Oncol.

[6] Smith I, Procter M, Gelber RD, Guillaume S, Feyereislova A (2007). 2-year follow-up of trastuzumab after adjuvant chemotherapy in HER2-positive breast cancer: a randomised controlled trial.. Lancet.

[7] Crone SA, Zhao YY, Fan L, Gu Y, Minamisawa S (2002). ErbB2 is essential in the prevention of dilated cardiomyopathy.. Nat Med.

[8] Slamon DJ, Leyland-Jones B, Shak S, Fuchs H, Paton V (2001). Use of chemotherapy plus a monoclonal antibody against HER2 for metastatic breast cancer that overexpresses HER2.. N Engl J Med.

[9] Yarden Y, Sliwkowski MX (2001). Untangling the ErbB signalling network.. Nat Rev Mol Cell Biol.

[10] Tao RH, Maruyama IN (2008). All EGF(ErbB) receptors have preformed homo- and heterodimeric structures in living cells.. J Cell Sci.

[11] Bersell K, Arab S, Haring B, Kuhn B (2009). Neuregulin1/ErbB4 signaling induces cardiomyocyte proliferation and repair of heart injury.. Cell.

[12] Grant S, Qiao L, Dent P (2002). Roles of ERBB family receptor tyrosine kinases, and downstream signaling pathways, in the control of cell growth and survival.. Front Biosci.

[13] Roux PP, Blenis J (2004). ERK and p38 MAPK-activated protein kinases: a family of protein kinases with diverse biological functions.. Microbiol Mol Biol Rev.

[14] Richard DE, Berra E, Gothie E, Roux D, Pouyssegur J (1999). p42/p44 mitogen-activated protein kinases phosphorylate hypoxia-inducible factor 1alpha (HIF-1alpha) and enhance the transcriptional activity of HIF-1.. J Biol Chem.

[15] Tennant DA, Frezza C, Mackenzie ED, Nguyen QD, Zheng L (2009). Reactivating HIF prolyl hydroxylases under hypoxia results in metabolic catastrophe and cell death.. Oncogene.

[16] Song G, Ouyang G, Bao S (2005). The activation of Akt/PKB signaling pathway and cell survival.. J Cell Mol Med.

[17] Dimmeler S, Fleming I, Fisslthaler B, Hermann C, Busse R (1999). Activation of nitric oxide synthase in endothelial cells by Akt-dependent phosphorylation.. Nature.

[18] Aki T, Mizukami Y, Oka Y, Yamaguchi K, Uemura K (2001). Phosphoinositide 3-kinase accelerates necrotic cell death during hypoxia.. Biochem J.

[19] Jiang Z, Zhang Y, Chen X, Lam PY, Yang H (2002). Activation of Erk1/2 and Akt in astrocytes under ischemia.. Biochem Biophys Res Commun.

[20] Favara BE, Franciosi RA (1972). Diphtherial myocardiopathy.. Am J Cardiol.

[21] Tanaka N, Masamura K, Yoshida M, Kato M, Kawai Y (2002). A role of heparin-binding epidermal growth factor-like growth factor in cardiac remodeling after myocardial infarction.. Biochem Biophys Res Commun.

[22] Iwabu A, Murakami T, Kusachi S, Nakamura K, Takemoto S (2002). Concomitant expression of heparin-binding epidermal growth factor-like growth factor mRNA and basic fibroblast growth factor mRNA in myocardial infarction in rats.. Basic Res Cardiol.

[23] Ushikoshi H, Takahashi T, Chen X, Khai NC, Esaki M (2005). Local overexpression of HB-EGF exacerbates remodeling following myocardial infarction by activating noncardiomyocytes.. Lab Invest.

[24] Liu X, Gu X, Li Z, Li X, Li H (2006). Neuregulin-1/erbB-activation improves cardiac function and survival in models of ischemic, dilated, and viral cardiomyopathy.. J Am Coll Cardiol.

[25] Nielsen LB, Perko M, Arendrup H, Andersen CB (2002). Microsomal triglyceride transfer protein gene expression and triglyceride accumulation in hypoxic human hearts.. Arterioscler Thromb Vasc Biol.

[26] Claycomb WC, Lanson NA, Stallworth BS, Egeland DB, Delcarpio JB (1998). HL-1 cells: a cardiac muscle cell line that contracts and retains phenotypic characteristics of the adult cardiomyocyte.. Proc Natl Acad Sci U S A.

[27] Andersen CL, Jensen JL, Orntoft TF (2004). Normalization of real-time quantitative reverse transcription-PCR data: a model-based variance estimation approach to identify genes suited for normalization, applied to bladder and colon cancer data sets.. Cancer Res.

[28] Schooler K, Wiley HS (2000). Ratiometric assay of epidermal growth factor receptor tyrosine kinase activation.. Anal Biochem.

[29] Elenius K, Corfas G, Paul S, Choi CJ, Rio C (1997). A novel juxtamembrane domain isoform of HER4/ErbB4. Isoform-specific tissue distribution and differential processing in response to phorbol ester.. J Biol Chem.

[30] Pouyssegur J, Lenormand P (2003). Fidelity and spatio-temporal control in MAP kinase (ERKs) signalling. Eur J Biochem 270: 3291–3299.. 3707 [pii].

[31] Lee KF, Simon H, Chen H, Bates B, Hung MC (1995). Requirement for neuregulin receptor erbB2 in neural and cardiac development.. Nature.

[32] Downward J (2004). PI 3-kinase, Akt and cell survival.. Semin Cell Dev Biol.

[33] Mebratu Y, Tesfaigzi Y (2009). How ERK1/2 activation controls cell proliferation and cell death: Is subcellular localization the answer?. Cell Cycle.

[34] Rohrbach S, Niemann B, Silber RE, Holtz J (2005). Neuregulin receptors erbB2 and erbB4 in failing human myocardium – depressed expression and attenuated activation.. Basic Res Cardiol.

[35] Ky B, Kimmel SE, Safa RN, Putt ME, Sweitzer NK (2009). Neuregulin-1 beta is associated with disease severity and adverse outcomes in chronic heart failure.. Circulation.

